# GLP‐1 Receptor Agonists and Weight Loss: A Critical Review of Mechanisms

**DOI:** 10.1111/obr.70148

**Published:** 2026-05-05

**Authors:** Giorgia Rutigliani, Tzu‐Wen L. Cross, Erica de Jong, Emily S. Glover, Ivan D. Mendoza, Mojisola Adisa, Keigo Tomoo, Xinyi Zhou, Richard D. Mattes

**Affiliations:** ^1^ Department of Nutrition Science Purdue University West Lafayette Indiana USA; ^2^ Department of Food Science Purdue University West Lafayette Indiana USA; ^3^ Department of Human Development and Family Science Purdue University West Lafayette Indiana USA

**Keywords:** appetite, food cravings, food noise, gastric emptying, microbiome, sensory function

## Abstract

GLP‐1 receptor agonist medications have the potential to promote marked weight loss, but maximal and sustained benefit may be compromised by poor compliance and cessation of use. Development of next‐generation medications that address current obstacles to effective use and development of effective adjunct treatments will benefit from better characterization of their mechanisms of action. This narrative review critically assesses eight purported mechanisms including modulation of appetite, chemosensory function, cravings/aversions, food noise, gastric emptying, the microbiome, incretin activity, and energy expenditure. Current evidence does not support a single dominant mechanism; a combination of subtle effects may underlie the efficacy of these medications. However, as experience with these medications and methods to assess their effects grows, it should be possible to better determine the relative importance of these and possibly other mechanisms.

## 1 Introduction

The marked increased trajectory of overweight/obesity prevalence in the US in the late 1970s [1, 2, 3, 4] prompted efforts to mitigate the problem, and it remains a high priority for research, clinical, public health, and regulatory communities [5, 6]. A focus on the gut and endocrine control mechanisms arguably stems from the seminal observation by Gibbs et al. that cholecystokinin (CCK) exerts anorectic effects in rats independent of malaise [7]. Considerable subsequent work revealed a wide array of gut hormones with pleiotropic effects, including an often weak and inconsistent [8, 9] impact on appetite and food intake. However, subsequent work with supraphysiological concentrations of selected gut peptides and administration of agonists for their receptors, most notably glucagon‐like peptide‐1 receptor agonists (GLP‐1RA), revealed that they were effective for weight management [10, 11, 12, 13].

The marked improvement in nonsurgical treatment efficacy has resulted in expanding use of these medications [14, 15]. A nationwide analysis identified roughly more than a million new GLP‐1RA users between 2011 and 2023 in the United States. From 2018 to 2023, annual spending on these medications increased from $13.7 billion to $71.7 billion [16]. In 2023, 89% of spending was for medications approved for the management of type 2 diabetes, and 11% for obesity [17]. However, there is a trend for greater use to manage overweight/obesity [18]. As of May 2024, approximately 12% of the population has used a GLP‐1RA medication, and about 40% of individuals report doing so to lose weight [19].

Despite the unprecedented level of success in nonsurgical moderation of body weight, estimates of discontinuation of medication use are high, varying between 20% [20] and > 50% [21]. Moreover, among patients who continued GLP‐1 RA therapy, only 48.6% achieved adequate adherence (medication possession ratio > 0.80) at 1 year, indicating that most had gaps in medication coverage exceeding 20% of days [20]. Both of these problems are greater in populations using these medications in nonclinical trial settings [22], which is concerning since treatment discontinuation often results in weight regain [23]. These poor adherence statistics underscore the importance of better characterizing and addressing undesirable treatment side effects to enhance compliance and identifying the most important mechanisms of action to guide future drug development [24]. Moreover, high treatment attrition has prompted thinking about ways to mitigate weight regain following for‐cause or planned withdrawal through behavioral and pharmacological approaches [25, 26]. These recommendations also require improved knowledge of the mechanisms by which these medications work as they seek to complement or replace their modes of action. Additionally, these medications have been reported to improve psychotic disorders, seizures, neurocognitive conditions (including Alzheimer's disease and dementia), coagulation and cardiometabolic disorders, infectious illnesses, and several respiratory illnesses or worsen other health conditions including gastrointestinal disorders, hypotension, syncope, arthritic complications, nephrolithiasis, interstitial nephritis and drug‐induced pancreatitis [24]. Realizing these benefits and/or reducing risks will also necessitate expanded understanding of the mode of action of GLP‐1RA medications.

Multiple mechanisms of action related to GLP‐1RA have been identified [27, 28]. Here, we critically assess the role and magnitude of eight commonly cited mechanisms. These include effects on appetite, chemosensory function, food cravings/aversions, food noise, gastric emptying (GE), the gut microbiome, incretin activity, and energy expenditure.

## 2 The Effect of GLP‐1 RAs on Appetite Regulation

Though the appetitive effects of endogenous GLP‐1 are limited under physiological conditions [29, 30, 31] and may not play a dominant role in modulating energy intake [9], supraphysiological concentrations of GLP‐1 have been shown to reduce hunger and augment fullness [32]. It is widely held that GLP‐1RAs promote weight loss through modulation of appetitive sensations. However, changes in subjective appetite ratings can be statistically significant but still small in absolute terms. Given the larger evidence base for this mechanism than most others, it is possible to present medication‐specific findings.

### 2.1 Liraglutide

Liraglutide is a once‐daily, fatty‐acid–conjugated human GLP‐1 receptor agonist (half‐life ~13 h) indicated for type 2 diabetes and chronic weight management. A 52‐week randomized controlled trial (RCT) in adults with obesity compared participants receiving liraglutide (3 mg/day) paired with intensive behavioral therapy (IBT) to those receiving IBT alone [33]. Hunger was reduced and fullness increased at weeks 6 and 24 for the individuals receiving liraglutide plus IBT compared to those receiving IBT alone, but these effects were not sustained from week 24 to the end of the intervention. A crossover trial investigating once‐daily subcutaneous liraglutide (1.8 or 3 mg) in individuals with obesity but without diabetes reported that during the 300‐min postprandial period, both 1.8 and 3.0 mg doses of liraglutide elicited a modest, equivalent rise in the overall appetite score, a composite of satiety, fullness, inverse hunger, and inverse prospective food consumption, indicating enhanced satiety with no dose‐dependent effect [34]. At no single time point did the appetite responses of the active and placebo‐treated participants differ significantly. Another study compared liraglutide with lixisenatide, a short‐acting GLP‐1RA, in adults with type 2 diabetes and found that both treatments produced comparable reductions in total and macronutrient intake and similarly suppressed subjective appetite ratings over 10 weeks [35].

In a year‐long, per‐protocol analysis, liraglutide 3 mg/day preserved postprandial appetite suppression (0% change vs. −14% with placebo; *p* = 0.023), but produced no significant changes in emotional‐ or uncontrolled‐eating scores compared with placebo. Because all outcomes were assessed only in participants who completed the protocol, these results may overstate the drug's true effectiveness relative to an intention‐to‐treat analysis [36]. Overall, these findings suggest that liraglutide produces only a modest, nondose‐dependent appetite effect that appears to be transient.

### 2.2 Semaglutide

Semaglutide is a once‐weekly, acylated human GLP‐1 receptor agonist (half‐life ~165 h) indicated for type 2 diabetes and chronic weight management. Semaglutide has been associated with a dose‐dependent reduction in self‐reported hunger and increased satiety, though the absolute differences in appetite ratings are often small. In one 12‐week study [37], subcutaneous injection of semaglutide was titrated in 4‐week increments, from 0.25 mg (weeks 1–4) to 0.5 mg (weeks 5–8) and finally to 1.0 mg (weeks 9–12), resulting in a significantly greater appetite suppression compared to placebo at 5 weeks (*p* = 0.0023). As with the previously cited Liraglutide study [34], this was observed as an overall effect though at no measured time point did the appetite ratings of the active and placebo treated participants differ significantly.

In a 20‐week randomized trial, weekly injections of 2.4 mg semaglutide in individuals with obesity improved postprandial appetite ratings (reduced hunger and prospective consumption; increased fullness and satiety; all *p* < 0.02) [38]. Oral administration of semaglutide, with doses ranging from 3 to 14 mg, in a cross‐over trial with individuals with type 2 diabetes mellitus (T2D), revealed that the drug led to improved satiety, hunger, and overall appetite score after a fat‐rich breakfast, but had no effect after a standard meal (breakfast) [39, 40].

Taken together, semaglutide produces consistent but often small absolute shifts in subjective appetite ratings in controlled settings that may be dependent on meal composition.

### 2.3 Dulaglutide

Dulaglutide is a once‐weekly, Fc‐fusion human GLP‐1 receptor agonist (half‐life ~90 h) approved for type 2 diabetes management. The effect of dulaglutide on appetite suppression is weaker compared to other GLP‐1 RAs. A 6‐month study in individuals with T2D receiving weekly subcutaneous injections of 1.5 mg dulaglutide revealed a 55% reduction in appetite vs. 12% in the control group at day 30, along with significantly improved fullness and satiety in the first week. However, appetite gradually returned to baseline levels after 3 months and remained unchanged for the duration of the study, suggesting an adaptive response over time [39].

### 2.4 Tirzepatide

Tirzepatide is a once‐weekly, dual GIP/GLP‐1 receptor agonist peptide (half‐life ~120 h/5 days) approved for type 2 diabetes and chronic weight management. Multiple trials have documented that tirzepatide suppresses appetite and, in some cases, to a greater extent than mono‐agonists (e.g., liraglutide) (*p* < 0.05) [40]. Other work suggests comparable effects to semaglutide. In one 28‐week trial, both tirzepatide (15 mg/week) and semaglutide (1 mg/week), administered in separate treatment arms, each reduced appetite compared to placebo (*p* < 0.001) in individuals with T2D, but there was no statistically significant difference in appetite suppression or in energy‐intake reduction between the tirzepatide and semaglutide groups [41]. Appetite suppression with both GLP‐1RAs was evident by week 4 and increased over time, remaining significantly lower than placebo through week 28. Additionally, separate researchers [42] observed a significant reduction in hunger perception with 10 and 15 mg tirzepatide, as well as a significant increase in postprandial fullness across all doses of tirzepatide (5, 10, 15 mg) and dulaglutide (0.75 mg), in a 52‐week trial involving 48 Japanese patients with T2D. Fullness did not differ across tirzepatide doses, and the greater effects observed at higher doses were primarily due to lower ratings in the control groups. Thus, tirzepatide enhances satiation modestly perhaps to a greater extent than liraglutide and comparably to semaglutide, but not in a dose‐dependent manner within the therapeutic range [43, 44, 45].

### 2.5 Effects on Different Populations

#### 2.5.1 Individuals With and Without Diabetes

GLP‐1RA effects on appetite vary based on metabolic status. Many studies [35, 39, 41, 42, 46] report a significant decrease in hunger and an increase in fullness in individuals with T2D or type 1 diabetes mellitus (T1DM) following liraglutide (1.8 mg/day) [47]. In contrast, one study noted semaglutide did not significantly affect hunger in a population without diabetes [37], while another study in a sample without diabetes observed a significant but modest increase in overall appetite score, satiety, and fullness, along with a reduction in hunger [34]. These findings are inconsistent but suggest a stronger effect on patients with diabetes.

#### 2.5.2 Individuals With and Without Obesity

Most studies investigating GLP‐1RAs and appetite have been conducted in individuals with obesity, limiting the ability to draw comparisons with leaner populations. As such, it remains unclear whether baseline adiposity modifies responsiveness to GLP‐1RAs. Interestingly, evidence from a study using a supraphysiological GLP‐1 infusion in 20 healthy, normal‐weight men showed significant effects on satiety, hunger, and fullness [32] demonstrating that lean individuals can respond robustly to GLP‐1 under certain conditions. Given increasing use of GLP‐1RAs by individuals with lower levels of adiposity [32, 48], further research is needed to determine whether the degree of adiposity alters the efficacy of GLP‐1RAs on appetite suppression.

In summary, GLP‐1RAs reduce subjective measures of appetite by roughly 0%–20% and lower ad libitum energy intake by up to ~35% [38, 41, 49]. However, appetite differences are often small in absolute terms at any single postprandial timepoint and a clear dose–response relationship is lacking [34, 49]. The error terms of measured appetitive sensations are generally small [34, 37] suggesting a lack of high and low responder subgroups. Diet composition may also influence appetite. For instance, semaglutide has shown greater effects on appetitive sensations following a fat‐rich meal compared to a standard test meal [46]. Such variability may partially explain the heterogeneity noted across studies. Finally, while some studies report initial appetite suppression, these effects may not persist with long‐term use. Diminishing changes in appetite scores over time, particularly with liraglutide and dulaglutide, suggest an adaptive response that limits sustained benefit [33, 36, 39]. Typically, drug effects peak within the first few days after dosing (e.g., semaglutide and dulaglutide peak concentrations occur ~1–3 days and ~24–72 h postdose, respectively), which makes within‐week variation in appetite plausible [44, 45]. Because trials assess appetite on a limited number and pattern of test days, the magnitude and time course of within‐week fluctuations in subjective appetite have yet to be quantified. The effects of these medications on satiation (within‐meal sensations) are also not known but may yield useful mechanistic information. Collectively, despite clinical reports of large shifts in appetite, measurements under controlled conditions suggest the need for caution in assuming that GLP‐1RAs produce clinically meaningful or lasting effects on appetite regulation.

## 3 Effects of GLP‐1RA on Chemosensory Function

Recent evidence suggests that signaling mechanisms between the oronasal region and peripheral tissues form an integrated system that optimizes the coordination of ingestive behavior, digestion, nutrient absorption, and metabolic regulation [50]. There is a strong theoretical basis for expecting GLP‐1RAs to alter gustatory and olfactory function and for these sensory systems to modulate metabolic processes. Reports of altered chemosensory function are not common among those using GLP‐1RAs, but unconscious, gradual shifts may hold implications for treatment compliance and efficacy.

Expanding beyond the oral cavity, nutrient‐sensing mechanisms in the gut utilize G‐protein‐coupled receptors (GPCRs) homologous to those found on the tongue. These receptors detect “taste” compounds that initiate intracellular signaling cascades that regulate nutrient transporter expression, facilitate nutrient absorption, and stimulate the secretion of enteroendocrine hormones, including GLP‐1, GIP, CCK, peptide YY (PYY), ghrelin, and secretin. These processes are important for maintaining energy balance and glucose homeostasis [51, 52].

### 3.1 Insulin Signaling and Taste Perception: Metabolic and Neural Links

GLP‐1‐induced insulin secretion may indirectly affect taste. In rodents, insulin regulates taste bud maintenance, with metabolic dysregulation diminishing receptor sensitivity [53]. In humans, direct evidence of insulin's role in taste bud maintenance is limited. However, some studies have reported that insulin is transcribed and translated in mammalian taste bud cells, indicating a potential local role in taste function [53, 54]. Additionally, impaired taste is well documented in individuals with diabetes. This may be due to altered insulin signaling or neuropathy [55]. Early work suggested a glucose‐specific impairment of taste in individuals with a family history of diabetes, supporting a hormonal or metabolic influence on taste, independent of neuropathy [56].

### 3.2 GLP‐1 and Taste Modulation: Insights From Rodent Models

Recent biophysical evidence from rodent studies suggests that GLP‐1 plays a significant role in modulating taste perception. GLP‐1 is expressed in taste buds and modulates taste via paracrine signaling to adjacent nerve fibers, a process regulated by dipeptidyl peptidase‐4 (DPP‐4). Increased DPP‐4 expression in diabetic models links metabolic dysregulation to altered taste perception, particularly reduced sweet sensitivity, potentially influencing food preferences [50, 57].

GLP‐1 receptor knockout mice exhibit reduced sensitivity to sweet compounds [51, 52, 58] and umami stimuli [58]. There is also evidence of a modest decrease in citric acid sensitivity in mice, indicating a potential role for GLP‐1 in sour taste function [52]. GLP‐1 receptor knockout mice also exhibit higher detection thresholds for dietary lipids, suggesting a reduction in oral fat sensitivity [51]. This effect may be mediated by lipid‐sensing receptors such as CD36 and GPR120, which contribute to fatty acid detection through distinct, concentration‐dependent signaling pathways and play key roles in oral fat perception and fat‐based food attraction [59, 60]. In contrast, no significant differences have been observed for bitter or salty taste sensitivity in the absence of GLP‐1 signaling [51] indicating quality‐specific effects. However, sodium intake has been shown to increase postprandial GLP‐1 secretion, likely through SGLT1‐mediated uptake [61]. Interestingly, although GLP‐1 receptor activation suppresses saline intake in rodents, there is no direct evidence that it alters salt taste perception [62].

### 3.3 GLP‐1RA and Taste Modulation in Humans

There are inconsistent data from human studies suggesting GLP‐1RAs alter taste. Some data indicate that liraglutide enhances sweet taste sensitivity (i.e., decreased detection thresholds). In contrast, a recent study evaluated taste quality recognition in GLP‐1RA users in a two‐phase design: in open‐label Phase 1, participants exhibited reduced identification of sweet, salty, sour, bitter, and umami tastes (fat was not assessed), and in blinded Phase 2, when subjects were unaware the study was evaluating taste changes, objective deficits persisted despite no self‐reported alteration in taste function [63]. Separately, diminished hedonic responses (i.e., subjective pleasure and liking) to sweet and high‐fat foods have been observed in adults with obesity treated with semaglutide [37] as well as in individuals with type 2 diabetes treated with liraglutide [64]. Variability in outcomes may stem from several factors. One key consideration is the type of GLP‐1RA used. Semaglutide has a substantially longer half‐life and enhanced ability to engage central appetite‐regulating pathways compared to liraglutide. It may elicit a more prolonged or potent modulation of taste‐related brain circuits [65]. Second, treatment duration and dosage may influence whether peripheral [66] or central [67] mechanisms dominate. Shorter treatments may primarily affect gut‐brain signaling, whereas longer treatments might alter central hedonic processing. Third, individuals with T2D often show altered taste sensitivity at baseline either masking or exaggerating treatment effects. Finally, variability in methodological approaches, including the specific taste dimension assessed, such as detection thresholds versus hedonic responses, further hampers interpretation and comparison across studies.

### 3.4 Olfaction

Olfaction plays a crucial role in food appeal and ingestive behavior through central and peripheral (gut olfactory receptors) pathways that regulate appetite and metabolism [68, 69].

#### 3.4.1 GLP‐1 and Insulin Signaling in the Olfactory Bulb

Although hormonal signaling molecules are not classical ligands for olfactory receptors, emerging evidence suggests that hormones such as insulin, GLP‐1, and ghrelin can modulate olfactory pathways, potentially influencing receptor expression and/or function [70, 71, 72]. The olfactory bulb experiences the highest concentration of insulin and contains the highest concentration of insulin receptors in the brain, suggesting it may play a significant role in insulin‐mediated neural function [73, 74]. Insulin influences olfactory responsiveness in distinct ways depending on its site of release and action. In humans, peripheral short‐term hyperinsulinemia reduces olfactory sensitivity in healthy individuals, potentially serving as a satiety signal that suppresses food‐seeking behavior [66]. In contrast, intranasal administration of insulin, which bypasses the periphery and directly targets the brain, enhances odor perception [67].

Given that GLP‐1RAs augment central insulin concentrations, they may contribute to improved olfactory function [75]. The olfactory bulb also expresses GLP‐1 receptors, as demonstrated in rodent studies [70, 76]. In this region, GLP‐1‐producing neurons enhance the excitability of mitral cells, key relay neurons in the olfactory circuit, by modulating voltage‐dependent potassium channels [77, 78]. Increased excitability of mitral cells likely amplifies the transmission of olfactory signals to higher brain regions involved in odor perception and feeding behavior, providing a pathway by which metabolic signaling molecules like GLP‐1 can influence sensory‐driven ingestive responses [77]. Behaviorally, GLP‐1 signaling in the olfactory bulb modulates olfactory‐driven foraging: blocking GLP‐1 receptors suppresses food‐seeking in lean mice, while activating them restores this behavior in obese mice [70]. Thus, while GLP‐1RAs elevate peripheral insulin, which may reduce olfactory sensitivity, their potential to enhance central insulin signaling and directly activate GLP‐1 receptors in the olfactory bulb suggests a nuanced, site‐dependent influence on olfactory‐driven ingestive behavior. More studies are needed to investigate whether GLP‐1RAs influence olfactory perception in humans and, if so, to delineate the relative contributions of peripheral insulin, central insulin signaling, and direct GLP‐1 receptor activation in olfactory brain regions.

#### 3.4.2 Peripheral Effects of Endogenous GLP‐1 on Olfaction

Olfactory receptors are part of the GPCR superfamily and have been identified in peripheral tissues, including adipose tissue, gut, pancreas, muscle, and brain [79, 80]. In these sites, they detect various metabolic signals, including short‐chain fatty acids (SCFA), ketone bodies, amino acid metabolites, and lipid‐derived molecules such as medium‐chain fatty acids. Through such signals, they modulate key metabolic processes, including insulin and glucagon secretion, fatty acid oxidation, lipogenesis, and thermogenesis [80, 81]. Similar to oral‐gastrointestinal signaling, olfactory receptors in the gut, such as OR51E1, play a role in GLP‐1 secretion, linking olfactory stimuli to peripheral metabolism [82]. These receptors are locally activated on enteroendocrine L cells by odorant ligands present in the gut lumen. Oral administration of specific odorants, such as nonanoic acid, enhances GLP‐1 secretion and lowers blood glucose concentrations in rats [83]. In vitro studies demonstrate that certain volatile compounds, such as diacetyl, can suppress GLP‐1 production and secretion in enteroendocrine cells [84]. This suggests that odorant‐mediated modulation of GLP‐1 occurs through local gut receptor activation.

#### 3.4.3 Effects of GLP‐1RAs on Olfaction

Impaired olfactory function is common in individuals with prediabetes and diabetes, and treatment with liraglutide enhances olfactory‐related brain activity and improves odor sensitivity [75, 85]. However, other studies report minimal or no effects of GLP‐1RAs on olfactory performance [63]. These discrepancies may be partly influenced by differences in study populations (e.g., with diabetes vs. without), types of GLP‐1RAs used, treatment duration, or variability in olfactory testing methods. Further research is needed to clarify the conditions under which GLP‐1 signaling influences olfactory function. Evidence suggests that GLP‐1 and insulin may influence olfactory and taste function via local and systemic mechanisms and that GLP‐1 may be released via activation of olfactory and taste receptors in the gut, yet the extent to which these processes drive changes in food preferences and food/energy intake remains poorly characterized. Although some studies indicate that GLP‐1RAs enhance sweet and fat taste sensitivity and improve olfactory function, others report minimal or inconsistent effects, raising questions about individual variability, methodological discrepancies, and the broader implications of these findings. Additionally, much of the current evidence relies on rodent models that may not fully capture human metabolic and behavioral responses. Further, the limited number of human studies often suffer from small sample sizes of individuals with varying health status administered different medications. Given the profound influence of taste and smell on ingestive behavior, a more mechanistic understanding of GLP‐1RA's role in sensory modulation is critical. Future research must disentangle the direct effects of GLP‐1RAs from secondary metabolic adaptations and establish whether these sensory changes are causally linked to appetite suppression and weight loss.

## 4 Effects of GLP‐1RA on Cravings/Aversions

It has been posited that GLP‐1RAs modify ingestive behavior through their influence on food cravings and aversions [86, 87], which are defined as motivations to consume or avoid specific foods that are sufficiently intense that they interfere with daily activities [88, 89]. This definition distinguishes cravings or aversions from appetitive sensations such as hunger, fullness, and desire to eat or hedonic responses. Cravings and aversions are typically assessed through self‐report methods, including questionnaires and food records. No standard or objective measurement index currently exists [90]. These responses can be triggered by external cues (e.g., social context, food appearance, or memories of past experiences) as well as internal states (e.g., hunger, stress, or fatigue). While cravings are not considered a disease and may not be inherently harmful, they can contribute to unhealthy dietary patterns and/or undesired weight gain in certain individuals [88, 91]. Some evidence suggests that cravings may predict future weight gain and that managing cravings supports long‐term weight loss maintenance [92]. Conversely, extreme, broad‐based aversions may lead to weight loss and/or nutritional deficiencies depending on the types and quantity of foods affected [90].

### 4.1 GLP‐1RA and Food Cravings

Several brain regions have been associated with reward‐seeking behaviors, mostly in the cortical areas in the mesolimbic reward system [93]. GLP‐1 receptors are expressed in these regions and have direct connections to the nucleus tractus solitarius (NTS) [94], potentially contributing to the regulation of hedonic eating [95]. GLP‐1RAs have also been linked to the regulation of dopaminergic neurotransmission, a contributor to reward processing [96]. Moreover, stress can influence food intake through cortisol‐dopamine interactions that may be influenced by GLP‐1 [97]. Regarding food aversions, anatomical and behavioral analyses reveal that GLP‐1 actions in the area postrema (AP) drive nausea and aversive responses [98]. Notably, administration of GLP‐1 is sufficient to induce a conditioned flavor aversion in rodents [99].

Evidence on the effects of GLP‐1RAs on food cravings remains limited, with even fewer studies exploring their contribution to food aversions. Some GLP‐1RA medications, such as lixisenatide and tirzepatide, reportedly suppress cravings. In one study, lixisenatide administration was associated with an inverse relationship between brain reward activity and self‐reported cravings and hunger among individuals who reduced their energy intake [100]. Similarly, an 18‐week study investigating the effects of tirzepatide in individuals with obesity reported significant reductions in cravings for sweets, carbohydrates, and fast food, though no changes were observed for cravings related to high‐fat foods or fruits and vegetables [91]. The extent to which these observed changes qualify as reduced cravings is uncertain. Commonly used craving measures primarily quantify how strongly an individual wants a specific food (urge intensity), whereas “food noise” reflects how often and how intrusively food‐related thoughts occur (cognitive preoccupation), which may or may not culminate in craving or eating. Distinguishing between the two may clarify whether reduced intake is due to appetite drive, food reward valuation, and/or disengagement from habitual cue‐triggered eating.

Preclinical studies suggest that Semaglutide can access areas of the brain involved in appetite and cravings regulation, particularly within hedonic neural pathways [101]. One of the most notable sets of investigations in this area is the STEP trials (Semaglutide Treatment Effect in People Living with Obesity), a series of randomized controlled trials investigating the effects of Semaglutide (2.4 mg/week) on cravings over a long time period (104 weeks) in individuals with overweight or obesity and weight‐related comorbidities but without diabetes. Participants receiving semaglutide demonstrated improved ability to resist cravings and control eating, as evidenced by their scores in the Control of Eating Questionnaire (COEQ). Specifically, cravings for savory foods showed sustained improvement throughout the study, while reductions in cravings for sweet foods improved up to 1 year but did not remain statistically significant over the full 2‐year period compared to the placebo. A systematic review reporting the effects of GLP‐1RAs on reward‐related behaviors indicated similar findings, with participants having less hunger and food cravings, better control of eating habits, and lower desire to eat high‐fat foods after Semaglutide injection compared to controls [37]. Similar to the STEP trials, reductions in savory food cravings appeared more robust than those for sweet cravings.

### 4.2 GLP‐1RA and Food Aversions

GLP‐1RAs may also promote specific food aversions. These aversions are often highly specific (e.g., to greasy or sweet foods) and are distinct from a general lack of appetite. While they may contribute to short‐term decreases in consumption of particular items, they are unlikely to account for the substantial weight loss associated with GLP‐1RAs, as they do not typically generalize across the diet. Existing research suggests that aversions are more likely to influence food preferences than total energy intake [102]. More recent qualitative studies involving patients taking GLP‐1RAs have noted these experiences anecdotally, particularly regarding high‐fat foods [37]. However, systematic research measuring the prevalence and dietary impact of aversions remains limited. Further exploration into the neurobiological and experiential differences between suppression of cravings and augmentation of aversions could help clarify their distinct roles in shaping dietary behavior under GLP‐1RA treatment.

In summary, no published study examining the effects of GLP‐1RAs has explicitly differentiated between cravings, food aversions, and more subtle appetitive sensations or hedonic responses. Instead, claims have been based on self‐reported data, often without training, terminology, or clear definitions with the assessment tools used. Thus, the true incidence and consequences of cravings and aversions related to GLP‐1RA use have yet to be identified, and existing evidence does not support a major independent contribution of these phenomena to GLP‐1RA‐induced weight loss. Nevertheless, cravings are an important and frequently overlooked aspect of long‐term weight management and should be considered in the context of GLP‐1RA therapy. These medications may help to suppress cravings, with a resultant contribution to weight loss and/or weight loss maintenance, though at this time the magnitude and consistency of effects appear limited. Additionally, there are inconsistent findings regarding the sensory characteristics of altered cravings (i.e., savory vs. sweet) with different medications that require verification. Even less clinical evidence is available regarding the incidence of GLP‐1RAinduced food aversions on weight management.

## 5 Effects of GLP‐1RA on Cognition (Food Noise)

Food noise refers to persistent, intrusive thoughts about food, with excessive food rumination often reported by individuals with obesity or certain eating disorders [103, 104]. In today's environment, where food is easily accessible and affordable, individuals who constantly think about food have increased opportunities to obtain and consume a surplus of energy [105]. It has been hypothesized that this easy access to food is a contributing factor in the current obesity pandemic [106].

Food noise is an internal phenomenon that may be elicited or amplified by external food cues. It may reflect heightened food cue reactivity, defined as conditioned physiological‐psychological response to environmental food cues. With respect to eating behaviors, the processing of external food noise could drive external eating. External eating, a concept guided by the externality theory of obesity [107, 108], describes how certain individuals (e.g., those with obesity) may be more inclined to eat in the presence of sensory food cues (e.g., sight or smell of food) [109]. As an internal manifestation, food noise does not necessarily require an external cue, but instead relies primarily on “persistent, intrusive thoughts about food stemming from interoceptive signals that are disruptive to daily life and make healthy behaviors difficult” [110]. Sources of internal cues consist of appetitive sensations as well as momentary, personal factors such as experiences of stress, hormonal change, and emotion [111]. Processing of internal food noise could be related to dietary disinhibition, defined as the tendency to overeat in response to various emotional (e.g., negative affect or mood) and habitual (e.g., cyclical and failed efforts to diet) factors [112, 113].

GLP‐1RA drugs may modulate food noise, a claim that has so far been supported primarily by anecdotal clinical observations stating that these drugs “quiet” the noise around food [114]. Interestingly, similar anecdotal reports are also made for diminutions of substance abuse (e.g., alcohol and nicotine) and compulsive behaviors (e.g., shopping and gambling) [115]. A clear physiological mechanism for this process has not yet been identified, but GLP‐1RAs may elicit changes in neural circuits mediating reward and aversion [109, 116]. Specifically, it has been hypothesized that the use of GLP‐1RAs diminishes one's food thoughts and related food‐securing actions to levels beyond those achieved by intensive behavioral interventions.

Several studies, including observational cohorts [117, 118, 119, 120, 121, 122, 123, 124], clinical trials [33, 36, 37, 40, 46, 47, 50, 125, 126, 127], and studies using neuroimaging methodology [48, 100, 128], have examined the effects of GLP‐1RAs on food noise and related eating behaviors. While study designs and populations varied, most included individuals with overweight/obesity [33, 36, 37, 40, 46, 47, 48, 50, 100, 117, 118, 119, 120, 121, 122, 123, 124, 125, 126, 127, 128], and many involved individuals with T2D. Some studies also included healthy participants [33, 36, 37, 40, 48, 117, 127] or those with conditions such as polycystic ovary syndrome [50] or T1DM [47]. Sample sizes and participant ages varied widely, but most research focused on adults aged 45 years and older. The majority of studies ranged from three to 6 months, with one observational cohort study following participants for 2 years [118]. Liraglutide and semaglutide were the most commonly studied GLP‐1RAs, though others, such as lixisenatide, dulaglutide, exenatide, and tirzepatide, were also examined. Comparators included placebo/saline injection [36, 37, 40, 46, 47, 48], behavioral therapy [33, 36, 127], other types of diabetes medications [121, 126, 128], and some trials had no comparator [50, 125]. Findings from these diverse methodological approaches are more similar than dissimilar and demonstrate that GLP‐1RAs produce the generalizable effects elaborated below. As the advancement of GLP‐1RAs continues with dual and tri‐agonists [129], it will be important to examine whether their use results in larger and sustained effects in reducing food noise. No study has measured food noise directly, given that validated tools for this construct have only recently emerged [110, 130]. Still, many studies assessed related eating behaviors, such as susceptibility to overeating in response to cues, using various questionnaires, such as the Three Factor Eating Questionnaire, the Dutch Eating Questionnaire, the Eating Inventory, the Control of eating Questionnaire, the 100 mm visual analog scale, and more. These collective questionnaires highlight both the conceptual relevance to food noise and the need for standardized, validated measures. Nevertheless, the measures featured in this analysis illustrate potential manifestations of food noise (e.g., maladaptive eating behaviors), that are proximal to weight management [131].

Most, but not all [120, 122], observational studies demonstrated significant reductions in external [119, 121, 123, 124] and uncontrolled eating [117] suggesting individuals may have experienced decreases in food noise over time. These findings are insightful, but limitations in study design prevent a clear attribution of changes of GLP‐1RA use to changes in external eating, which could be confounded by other factors, such as behavioral reactivity and lifestyle modification. Clinical trials are better suited to explore the modulating role of GLP‐1RAs on food noise. Two identified studies found significant decreases in external [125] and uncontrolled [1] eating; however, they did not include a comparator. More insightful are studies utilizing within‐subjects crossover designs or between‐subjects parallel/multi‐arm designs.

One of three crossover studies reported that daily injection of liraglutide led to a significant reduction in disinhibition compared to participants in the placebo group [47]. Other studies used the COEQ and reported a reduction in food thoughts and improved control of eating when using injected or orally administered semaglutide [37]. Notably, in one study [37], the VAS question “How often have you had thoughts of food” had the second highest mean decrease of all COEQ items, which probably aligns closest with the current conceptualization of food noise. Given the limited scope and number of studies, it is difficult to draw definitive conclusions on whether one drug outperforms another in reducing food noise.

Among parallel/multi‐arm studies, the GLP‐1RAs outperformed the standard control in decreasing dietary disinhibition [37, 40], binge eating [126], and food preoccupation [33]. However, one 12‐week study reported null effects for changes in uncontrolled eating with improvement in cognitive restraint [36], potentially due to the derivation of these findings from a secondary, exploratory analysis. Exploratory analyses are useful in generating hypotheses [132], but they should also be assessed with additional considerations to determine veracity when findings are used to explore causal processes [133]. Future studies should continue to incorporate a standard control group, and for these studies, sample sizes should be powered to examine food noise as a primary outcome.

Studies measuring brain activity through neural imaging techniques also provide important insights into food noise and the cognitive processing of food cues. Two studies reported that food cues, post‐GLP‐1RA administration, drive differential brain activation among individuals with obesity and individuals of normal weight. Individuals with obesity showed decreased responses in the amygdala and insula [48] and in the fusiform gyrus and lateral ventricle [100]. The amygdala and insula are areas in the brain proposed to help with appetite regulation, and they guide processes such as diminishing the value of food upon satiety [48]. Thus, decreased activity in these areas may relate to a lower emotional salience to food cues. The fusiform gyrus helps with object recognition and could be associated with the neural processing of visual food cues [100], so decreases in external eating over time could be due to diminished reward from visual food cues while taking these drugs. Another functional magnetic resonance imaging study argues eating behaviors could be a mechanistic explanation for individual variability in weight loss when on GLP‐1RAs [128]. This perspective is valuable and holds merit in short‐term weight loss studies, but given the potential for eating behaviors to longitudinally adapt with GLP‐1RA use, future work should examine change in food noise and eating behaviors over time.

Taken together, studies with stronger methodological designs suggest GLP‐1RAs contribute to diminished external and internal manifestations of food noise, as shown through changes in eating behaviors and resultant weight loss. Future studies should use direct measures of food noise, such as the recently published Food Noise Questionnaire [110] or Ro Allison Indiana Dhurandhar Food Noise (RAID‐FN) Inventory [130], to better understand its modulation by GLP‐1RA use.

## 6 Effects of GLP‐1RA on Gastric Emptying

It is commonly stated that a primary mechanism for weight loss with the use of GLP‐1RAs is augmented satiety due to delayed gastric emptying (DGE). GLP‐1RAs mimic the enterogastrone function of endogenous GLP‐1, which delays GE and inhibits gastric secretions [134, 135, 136]. However, DGE may not be the primary mechanism behind satiety and weight loss for several reasons. First, studies reporting statistically significant effects of GLP‐1RA usage on DGE and improved satiety vary greatly in methodology and study design, often employing inadequate gastric motility measurement techniques to form these conclusions [137, 138, 139, 140]. Second, it cannot be assumed that DGE leads to greater satiety, as studies show these factors are not tightly coupled [35, 38, 141, 142]. Finally, there is controversial evidence regarding whether the effect of DGE is strong enough to significantly reduce subsequent energy intake [35, 38, 143].

Both endogenous and exogenous GLP‐1 delay GE and reduce the frequency of the migrating motor complex (MMC) in the small intestine [139, 141, 144, 145]. This effect is noted in both healthy individuals and those with diabetes and appears to be dose‐related [34, 139, 141, 146, 147, 148]. GLP‐RAs mimic this property and may reduce GE by modulating antro‐pyloro‐duodenal motility via cholinergic pathways [136, 141, 149]. Others hypothesize that prolonged distention facilitates greater cumulative release of gastrointestinal hormones, thereby sustaining stimulation of gastrointestinal vagal receptors [148, 150, 151]. Similarly, GLP‐1RAs may continually stimulate GLP‐1 receptors on vagal afferent nerves, indicative of the presence of nutrients and thereby delay GE. As a result, chyme remains in the stomach, sustaining intragastric volume and prolonging activation of gastric mechanoreceptors, specifically in the antrum [139, 141, 147, 152]. The antrum is a source of satiation signals, and many studies report that GLP‐1 redistributes gastric contents, favoring the antrum [136, 139, 141, 153]. Some evidence suggests that this extended enhancement of satiation/satiety attributed to GLP‐1RA concentrations may reduce energy intake, ultimately resulting in weight loss [139, 141]. While the effect of GLP‐1 on GE is well‐documented, findings on the magnitude of this effect vary. Some studies report a substantive increase in GE time, while others conclude the impact is minimal [137, 143, 147, 154, 155]. Inconsistent findings may stem from differences in research design and methodologies. Studies report that GE can be significantly delayed in individuals who initially exhibit a rapid emptying rate, a characteristic of obesity and diabetes [137, 139, 141, 142]. Age, sex, and blood volume may also contribute to interindividual variations in baseline gastric‐emptying rates and skew results [38, 137, 141, 156, 157, 158]. Additionally, selection bias is possible where individuals with gastroparesis are less likely to participate in GLP‐1RA studies due to the potential for exacerbated symptoms [138].

Differences in gastric motility measurement techniques further complicate findings as no single method accounts for all variables affecting GE [141]. Gastric scintigraphy remains the “gold standard” for measuring GE. This technique directly and concurrently tracks both solid and liquid meal components from ingestion to excretion. While comparatively expensive, it is non‐invasive and provides accurate measurements of intragastric chyme distribution [141]. Other techniques, such as C‐breath tests, ultrasound, and D‐xylose plasma concentration measurements, are indirect and may only assess liquid emptying [137]. The acetaminophen absorption (AA) test, another common method, indirectly measures GE by tracking the appearance of paracetamol in the bloodstream once it reaches the small intestine. However, because acetaminophen is only absorbed in the small intestine rather than the stomach, this method assumes rapid absorption into the duodenum, which may not always be accurate [159]. Additionally, this test primarily measures the liquid phase of GE. Given these limitations, results from AA tests must be interpreted with caution [38, 139, 141].

Assuming GLP‐1RAs significantly delay GE, questions remain about whether the effects are clinically meaningful. A recent meta‐analysis compared pooled GE times in GLP‐1RA users and placebo controls [143]. While no significant difference was observed using AA tests, gastric scintigraphy studies reported a pooled mean difference of 40.2 min, concluding that this delay was not clinically meaningful. Similarly, a study using ^13^C‐breath test to determine the effect of liraglutide and lixisenatide on GE in those with T2D reported a delay of 25 ± 10 and 52 ± 17 min, respectively [35]. GE rates in people with obesity tend to be more rapid compared to healthy individuals, yet the reported delay is also not clinically meaningful. Furthermore, the correlation between DGE and appetite reduction or weight loss is weak [35, 141].

Many studies, even those with substantially different results for GE rates, do not report significant correlations between GE and satiety [38, 137, 142, 147, 148]. One study using gastric scintigraphy reported physiological (0.3 pmol·kg^−1^·min^−1^) and supraphysiological (0.9 pmol·kg^−1^·min^−1^) doses of GLP‐1 resulted in solid food retention in the antral region of the stomach for 60–120 and 75–120 min, respectively [147]. Despite this delay, there was no significant relationship between the amount of retained gastric content and appetitive sensations [147]. Similarly, a study using 10 mcg of lixisenatide reported DGE, but theorized a centrally mediated reduction in energy intake.

A study conducted on six men with obesity using AA testing determined that less than 50% of a mixed‐nutrient meal emptied within 180 min in individuals infused with GLP‐1 (0.75 pmol·kg^−1^·min^−1^), whereas the saline‐infused control group exhibited full GE within the same time frame [148]. This study reports a significant delay in GE rates and a significant reduction in hunger, desire to eat, and prospective food consumption 4 h post‐ingestion compared to the control group. However, feelings of fullness 4 h post‐ingestion were not significantly different between GLP‐1 and saline. Although this study demonstrates DGE by exogenous GLP‐1, the lack of a significant effect on feelings of fullness suggests these are not tightly coupled [148]. A similar study supports this concept. A RCT with 16 healthy men observed no statistically significant effects on hunger or fullness scores at GLP‐1 infusion concentrations of 0.375 and 0.75 pmol·kg^−1^·min^−1^. Even supraphysiological doses (1.5 pmol·kg^−1^·min^−1^) did not increase satiety ratings [154].

In a 20‐week study with parallel groups, one group received 21 progressively increasing doses of semaglutide and another placebo. Following adjustment for body weight, no difference in GE rates was observed using AA [38]. Despite using an AA test, the study was well‐designed to assess GLP‐1's impact on satiety. This study demonstrates that GLP‐1RA's satiety‐inducing property is observed when DGE is not.

Given these conflicting findings, it appears unlikely that the reported enhanced satiety and weight loss observed with GLP‐1RAs can be attributed to their effect on DGE. Satiety is governed by a complex interplay of hormonal and neural signals and modifying a single factor does not fully explain the mechanisms behind sustained satiety and reduced energy intake [150]. GLP‐1RAs' marked success in weight management cannot be primarily attributed to their mimicry of endogenous GLP‐1's enterogastrone property.

## 7 Effects of GLP‐1RA on the Gut Microbiome

The gut microbiome refers to the diverse community of microorganisms, including bacteria, viruses, fungi, and archaea, that reside in the gastrointestinal tract. These microbes play essential roles in digestion, immune function, and energy regulation, and they influence host metabolism, inflammatory responses, and even communication with the brain, profoundly impacting overall health and disease risk. In both rodent models and human studies, GLP‐1RA therapy induces measurable changes in gut microbial composition, which may in turn modulate microbial metabolic outputs and influence host physiology. For instance, the use of GLP‐1RA decreases the Firmicutes/Bacteroidetes ratio, which is a feature that has been associated with lean phenotypes [160]. GLP‐1RA has also been shown to promote the presence of beneficial bacteria, such as *Lactobacillus*, in the gut [23]. Changes in the gut microbiome at the community level have been reported, with GLP‐1RA promoting greater alpha‐diversity in adults with T2D [161] and diet‐induced obese mice [162]. However, these shifts may reflect not only direct drug‐microbe interactions but also changes secondary to reduced energy intake and altered gastrointestinal transit.

Perhaps the strongest causal evidence for the gut microbiome's involvement in GLP‐1RA‐induced metabolic regulation comes from antibiotic and microbiota transplantation studies. When treated with antibiotics to abolish their gut microbiome, GLP‐1RA‐treated mice had reduced weight loss, worse glycemic response, greater insulin resistance, and greater adipocyte size [160, 163]. Furthermore, germ‐free mice that received microbiota from GLP‐1RA‐treated diabetic mice showed improved glucose‐stimulated insulin secretion compared to those that received microbiota from untreated diabetic mice [160].

Other than reducing inflammation by promoting intestinal barrier function, additional mechanisms have been suggested by which the gut microbiome may mediate GLP‐1RA‐induced metabolic regulation, such as through the production of microbial metabolites. The gut microbiome regulates metabolism, appetite, and glycemic responses partly through influencing GLP‐1 production via a variety of metabolites it generates, such as SCFA [164, 165, 166] and bile acids [167, 168]. GLP‐1RA medications may alter the synthesis of these microbial metabolites. For example, GLP‐1RA medication enhanced the relative abundance of SCFA‐producing bacteria [169, 170] in diabetic rodents. While the reported data are limited, a functional impact may exist, as GLP‐1RA treatment increases fecal SCFA concentrations in humans with T2D. SCFA may augment satiety signaling by creating a potential positive feedback loop that amplifies the anorectic effects of GLP‐1 through activating colonic free fatty acid receptors FFAR2 and FFAR3 on enteroendocrine L cells and vagal afferents [171, 172, 173].

Primary bile acids are synthesized by the liver and are metabolized into secondary bile acids by the gut microbiota. Although data are scarce, GLP‐1RAs may modulate bile acid metabolism by increasing serum unconjugated deoxycholic acid and decreasing taurine‐conjugated deoxycholic acid [174]. Due to bile acids' potent role in regulating energy metabolism and inflammation, they have been speculated to contribute to GLP‐1RA‐induced metabolic regulation via microbiome‐mediated mechanisms. Some bile acids can stimulate insulin and GLP‐1 secretion through activation of the farnesoid X receptor (FXR) and TGR5 pathways [175].

Another proposed mechanism linking GLP‐1RA therapy and the microbiome is improved intestinal barrier function through promoting tight junction integrity and thereby reducing gut microbiome‐derived inflammation. Indeed, GLP‐1RA reportedly exerts anti‐inflammatory effects on inflammatory bowel diseases [176, 177, 178]. GLP‐1RA treatment improved intestinal permeability and reduced oxidative stress, intestinal and systemic inflammation, circulating endotoxin lipopolysaccharide (LPS) level, and pro‐inflammatory cytokine interleukin 6 in diabetic rats [179] and LPS‐challenged rats [180]. GLP‐1RA has also been shown to promote the relative abundance of the gut microbial taxon *Akkermansia muciniphila* , a species that increases with enhanced intestinal mucus production. A greater proportion of *A. muciniphila* reduces intestinal permeability and inflammation [162, 181, 182].

In summary, GLP‐1RAs alter the relative abundance of gut microbial species and enhance alpha diversity either through direct effects or secondary to medication‐promoted weight loss. Preliminary studies with antibiotics and microbial implants support a direct effect possibly through satiety signaling, reduced inflammation and/or modulation of bile acid metabolism. The degree to which these effects alter food intake and body weight remains uncharacterized.

## 8 Incretin Effects of GLP‐1RAs

GLP‐1RA administration promotes insulin secretion when blood glucose concentrations are elevated [183, 184], resulting in a reduction in glycemia. There is a long‐standing view that the sensation of hunger is intensified under conditions of elevated insulin and lower blood sugar concentrations [185]. Some studies focus more specifically on insulin [139, 186] and others on glucose [187, 188] as drivers of appetitive sensations. Still, other work indicates a lack of a causal relationship between postprandial glucose and/or insulin concentrations and subjective hunger [185, 189]. There is unquestionably a postprandial rise of insulin and a subsequent reduction of glucose, perhaps even below premeal concentrations, that is accompanied by a reduction of hunger. However, these associations are unlikely to reflect a causal relationship due to no effects on appetite observed in most studies [190, 191, 192] under controlled conditions of clamping glucose or insulin. Thus, it is questionable that the incretin effect of GLP‐1RA plays an important role in appetite modulation and thereby contributes to a reduction of food/energy intake and body weight.

Patients with diabetes have lower incretin effects than individuals with normoglycemia and tend to lose less weight on treatment. Data from The Semaglutide Treatment Effect in People with obesity (STEP) trials, which assessed the efficacy of semaglutide (2.4 mg) in patients with normoglycemia or T2D, indicated greater weight loss (average of 16.8% across STEP‐1, 3, and 5) in healthy patients with mean HbA1_c_ of 5.7% compared with a mean 9.64% weight loss in participants with HbA1_c_ of 8.1% (STEP 2 trial) [10, 193, 194, 195]. A similar observation was made in SURMOUNT‐1 and SURMOUNT‐2 trials, where similar doses of tirzepatide were administered to participants with obesity or overweight and normal to only prediabetes blood glucose concentrations (mean HbA1_c_: 5.6%, fasting glucose: 95.3 mg/L, fasting insulin: 86.4 pmol/L) and to people with T2D (mean HbA1_c_: 8.07%, fasting glucose: 161.2 mg/L, fasting insulin: 83.6 pmol/L), respectively, for 72 weeks. In SURMOUNT‐1, the percentages of weight reduction for participants with normal to slightly elevated glycemia was −20.9%, while changes in glycemia were −10.6 mg/dL (fasting glucose) and −0.51% (HbA1_c_) [196]. Values for glycemia and weight loss for SURMOUNT 2 were −48.9 mg/dL (fasting glucose), −2.07% (HbA1_c_) and −14.7% (tirzepatide, 15 mg) [197], respectively. In SURMOUNT 3 and 4, which recruited individuals with overweight or obesity and prediabetes or normoglycemia, body weight decreased by 18.4% and 26% respectively, while changes in HbA1_c_ were −0.5% and −0.57% [23, 195]. The glucose‐lowering effect in T2D is consistent with the incretin activity of GLP‐1RA but there is no effect observed in healthy participants. Moreover, treatment effects on insulin secretion were not reported in these trials although the proof‐of‐concept study for tirzepatide shows augmented insulin secretion in patients with diabetes [198]. Similarly, in another small cohort study, the administration of GLP‐1RA to individuals with or without diabetes reported enhanced insulin secretion in healthy individuals about three to five times more than it increased insulin secretion in individuals with diabetes [199]. Thus, these trials indicate reductions in glycemia were greater and weight loss was less in the patients with T2D compared to individuals with similar adiposity but glycemia in the normal or prediabetic range. Such findings are not consistent with a view that elevated insulin and lower blood sugar concentrations augment hunger and food intake resulting in greater adiposity.

It should also be noted that the direction of causality between incretin effects and weight loss is uncertain. One could posit that the greater insulinotropic effect of GLP‐1 in healthy individuals could explain why they tend to lose more weight compared to patients with diabetes placed on a similar dose of GLP‐1RA or GIP/GLP‐1RA. A recent consensus from post hoc studies that compared single and dual receptor agonists suggested that changes in bodyweight may be mostly responsible for improved insulin sensitivity in individuals with diabetes [200]. Consequently, loss of excessive fat mass may be playing an important role in restoring insulin sensitivity as opposed to the reverse. Taken together, available data from trials of single GLP‐1 and dual GLP‐1/GIP receptor agonists do not reveal that an incretin effect is responsible for weight loss in healthy individuals and those with T2D.

## 9 Effects of Endogenous GLP‐1 on Energy Expenditure

Another mechanism by which GLP‐1RAs may decrease body weight is through augmentation of energy expenditure. Assessments have been made of these drugs on resting and postprandial energy expenditure, the thermic effect of feeding, and physical activity.

The acute effects of GLP‐1 infusion on energy expenditure have been studied in diverse populations for nearly three decades. A systematic review reported that intravenous or subcutaneous GLP‐1 infusions do not increase basal or resting energy expenditure in humans [201]. A later study concurred and expanded the evidence base noting that GLP‐1 infusion during isoglycemic clamp did not affect resting energy expenditure [202]. Similarly, infusion of GLP‐1 does not increase postprandial energy expenditure [203]. Indeed, in some reports, postprandial energy expenditure was paradoxically suppressed by acute GLP‐1 infusion in individuals with a normal weight and in individuals with obesity [204, 205]. This may relate to the acute inhibition of GE by GLP‐1 [140, 205]. Additionally, this inconsistency may stem from a difference in measurement timing. For example, a decline was reported when energy expenditure was measured every hour for 4 h during the postprandial time period and the largest effect was noted in the first hour [204, 205]. In contrast, no effect was observed when measurements were only obtained before and 210 min after meal ingestion [203]. Additionally, the physical form of the meal was different between the studies. A reduction of energy expenditure was noted following ingestion of a solid meal, and no effect was reported with a liquid‐based meal. This may relate to the slower emptying of solid versus liquid loads [206]. Overall, the evidence indicates GLP‐1 administration leads to no effect or a decrease in thermogenesis. There is no evidence to support that GLP‐1 increases thermogenesis, as would be expected were it to contribute to weight loss.

### 9.1 The Effect of GLP‐1RA on Energy Expenditure

The evidence for the effect of acute GLP‐1RA injection on energy expenditure is limited, but suggests a lack of effect [207]. Chronic GLP‐1RA treatment effects have been variable. Exenatide [208, 209, 210] and liraglutide [139, 211, 212] had no significant impact on resting, total, and/or sleeping energy expenditure in some studies, while others have reported a reduction in total energy expenditure by exenatide [213] and liraglutide [34, 214]. Additional studies have been published, but they lacked an appropriate control group or calculated energy expenditure indirectly [215, 216, 217, 218]. Further, it is important to note that body weight, body composition, and age influence energy expenditure measurements [219], and chronic energy restriction itself lowers energy expenditure [220]. Many studies did not adjust for these potential confounders. Thus, if an individual experienced significant weight loss from the treatment, energy expenditure could be underestimated at the post‐intervention time point. However, even when adjustments were made for at least one of these factors, the results remained inconsistent; with one study reporting an increase of 12%–17% [221], two studies reporting no effect [37, 209], and one study noting a trivial (< 10%) decrease at an early time point (4–12 weeks) of the intervention [214]. Overall, the chronic effect of GLP‐1RA administration on energy expenditure does not support a substantive increase, indicating it is an unlikely mechanism for weight loss due to GLP‐1RA use.

Given that exogenous GLP‐1 might suppress diet‐induced thermogenesis, this raises the question of whether similar effects are observed with GLP‐1RA. One study reported that an acute exenatide injection had no effect on postprandial energy expenditure [207]. However, it should be noted that energy expenditure was measured before and 210 min after meal ingestion, similar to the study reporting no effect of exogenous GLP‐1 infusion on postprandial energy expenditure [203]. Chronic exenatide injection also appeared to have no effect on diet‐induced thermogenesis [216]. Overall, the current limited evidence indicates there is no observed effect of GLP‐1RA use on diet‐induced thermogenesis.

Recently, the effect of GLP‐1RA administration on physical activity has been assessed [213, 222], with some studies suggesting a potential increase [222] and others demonstrating no changes [213]. Whether any increase in physical activity is a direct effect of GLP‐1RA use or a secondary effect of weight loss with improved mobility or motivation is not known. In most studies, energy expenditure measurements are conducted under well‐controlled experimental settings that do not account for daily activities. As physical activity is the most variable component of energy expenditure [223], future studies should examine the behavioral effects of GLP‐1RA under naturalistic living conditions.

Most work on energy expenditure to date has focused on single agonists, but the increased weight loss efficacy of dual and tri‐agonists [224, 225] suggests they warrant additional study, especially those including glucagon. Glucagon infusion increases energy expenditure [226] and does so independently of brown adipose tissue thermogenesis in humans [227]. In rodents, a tri‐agonist increased energy expenditure via glucagon receptor agonism [228, 229]. However, early findings for dual GLP‐1, GIP, and glucagon receptor agonism in humans have revealed chronically lowered lean body mass‐adjusted total and resting energy expenditure [230]. Another study did not find an effect on total and resting metabolic rate, but sleeping metabolic rate was lowered by dual agonism [231]. The mechanisms of both acute and chronic effects of glucagon agonism on energy expenditure in humans remain to be better characterized.

In summary, there is a lack of evidence that GLP‐1RAs induce weight loss via increased energy expenditure. However, increased energy expenditure due to enhanced physical activity warrants further study. Future generations of GLP‐1RA drugs that aim to exploit this mechanism are being developed and may hold promise.

## 10 Conclusion

GLP‐1RA medications have led to unprecedented, nonsurgical levels of weight loss. There has been considerable speculation about the mechanisms underlying this therapeutic benefit. An improved understanding will aid new drug development, beneficial adjunct therapies, and, possibly, approaches to enhance drug use compliance and adherence. However, the evidence reviewed here (see Table 1 for a summary) does not appear consistent with the substantial and sustained 15%–25% weight loss achieved with these medications. It may be that there are other mechanisms not considered here that better account for the observed weight management benefits. Alternatively, the impact of the reviewed mechanisms could be underestimated due to insufficient understanding of their roles or that there is synergy whereby modest changes in multiple mechanisms support a larger impact on body weight.

**TABLE 1:** Summary of evidence across proposed mechanisms of glucagon‐like peptide‐1 receptor agonist–induced weight loss.

Mechanism	Magnitude	Consistency	Dose–response	Temporal persistence	Clinical relevance
Appetite/satiety	Statistically significant, but small to moderate effect sizes	Directionally consistent (↓ hunger/ ↑ satiety/fullness) across controlled trials, but heterogeneous by measure and context	Inconsistent within therapeutic ranges	Several reports effects are transient	Contributes to reduced energy intake and weight loss, but shifts alone are modest and likely do not fully explain clinical outcomes
Chemosensory function (taste and olfaction)	Modest effects with magnitude poorly quantified and inconsistent across endpoints (e.g., thresholds vs. hedonic responses)	Low: heterogeneous findings across studies, sensory domains (taste vs. smell), and outcome type (detection/recognition thresholds vs. scaling vs. hedonic response)	No evidence	Not established.	Uncertain but potentially meaningful: may contribute to changes in food liking/selection and adherence, but causal links to energy intake and weight loss remain unproven
Food cravings/aversions	Small to moderate. ‐Cravings: reduced; but effect sizes are rarely standardized and often quality‐specific (savory > sweet in some data). ‐Aversions: uncertain/likely small	Moderate for reduced cravings: more consistent signal to “resist cravings,” less consistent for specific craving categories across time. Low for aversions: inconsistent and under‐studied	Unclear/limited evidence	Cravings may attenuate over time for certain categories (e.g., sweet) while other qualities may persist (e.g., savory), but not reliably across studies	Potentially meaningful for adherence and weight maintenance; uncertain as an independent driver of weight loss
Cognition—food noise	Moderate (on proxy outcomes): across trials/observational work, reductions in external eating, uncontrolled eating, disinhibition, food preoccupation/thoughts about food are commonly reported, but effect sizes vary by instrument and context	Moderate to high (for proxy measures): most studies show improvements in cue‐driven eating/disinhibition/food thoughts	Unclear/insufficient evidence	Probably sustained over months, but time‐course and adaptation are not well characterized	Potentially high (behavioral adherence and real‐world eating control), but causality and construct validity are still developing
Gastric emptying	Modest and method‐dependent (high inter‐individual variability)	Low–moderate (high heterogeneity by measurement technique, baseline gastric emptying rate, population, and study design)	Evident in acute settings	Often attenuates over time	Low (delays are often not clinically meaningful, and correlations with satiety/weight loss are weak)
Microbiome	Moderate changes in composition/alpha‐diversity, but effects can be confounded by reduced intake, weight loss, and altered transit.	Moderate evidence of association with shifts in microbiome composition/diversity, but low consistency for specific taxa or microbial “signatures”	Unclear/insufficient evidence	Uncertain	Potential but not established
Incretin activity (insulin/glucagon)	High for glycemic control in T2D; minimal in normoglycemia. As a driver of appetite/energy intake, effects appear small/indirect	High consistency for insulinotropic + glucose‐lowering effects in T2D	Present for metabolic endpoints (greater receptor agonism tends to produce greater glucose lowering/insulin secretion within therapeutic ranges)	Sustained with continued treatment for glycemic outcomes	High clinical relevance for diabetes management and cardiometabolic risk via glycemic improvement; low relevance as a primary mechanism of weight loss/appetite regulation
Energy expenditure	None to small; often null or slightly decreased	Moderate–high consistency for “no meaningful increase” in humans	Not established for GLP‐1RAs as a class. Potential dose‐related effects for glucagon receptor agonism (dual/tri‐agonists)	Insufficient evidence	Low as a primary mechanism of GLP‐1RA weight loss

Abbreviations: GLP‐1RA, glucagon‐like peptide‐1 receptor agonist; T2D, type 2 diabetes.

Clarification of these alternatives should be a high priority for future research. As a base to build on, the present review finds reduced hunger and/or augmented fullness are documented, but of modest magnitude, not dose‐dependent, and appear transient over time. Subjective appetite ratings may be related to diet composition (e.g., high or low fat), vary by health status (e.g., obesity or diabetes), and are frequently assessed over short time windows; as such, they do not reliably capture real‐world eating patterns or long‐term effects on energy balance. Thus, present understanding of their role in weight management is incomplete. Subtle chemosensory shifts may also contribute: gradual and often unnoticed changes in taste or smell can still alter hedonic valuation and food choice over time. This principle is consistent with population‐level reformulation strategies, which rely on incremental reductions that consumers typically do not detect while preferences adapt (e.g., the UK salt‐reduction program [232]). Consistent with this, a recent study reported that objective chemosensory testing can reveal changes not captured by self‐report [63], underscoring that “not noticing” does not imply “no effect.” Cravings and aversions generally hold limited impact on body weight, due, in part, to their specificity and little evidence suggests this is markedly altered by GLP‐1RA medications. Food noise is a new concept that remains poorly characterized and, until recently, without an agreed‐upon method of measurement. However, there are consistent clinical reports of GLP‐1RA‐induced diminutions of persistent, intrusive food thoughts that may underlie excessive energy intake. This may still prove to be a dominant mode of action. If so, it will be critical to characterize its sustainability and what adjunct treatments will be required to minimize this driver with cessation of GLP‐1RA use. Delayed GE is also a consistent finding, but of short duration (e.g., ~40 min) and poorly correlated with appetitive ratings, the presumed mediator of reduced energy intake and body weight. Enhanced fiber intake has been proposed to augment GLP‐1RA effects on GE and GI transit, but given complications of diarrhea and constipation with medication use, careful attention to the types of fibers ingested will be required to ensure symptoms are not intensified and possible beneficial effects related to slowed emptying/transit are not undermined. There are suggestive effects that GLP‐1RAs may alter the gut microbiome with resultant augmentation of appetitive sensations, but given the evidence on appetite indicates it is of limited magnitude, and transient, the importance of this mechanism is uncertain. GLP‐1RA effects on the microbiome may hold greater clinical importance for other health conditions. This review did not find evidence of a direct incretin effect or an increase of any dimension of energy expenditure on weight loss or maintenance. It may be that the constellation of multiple subtle effects is sufficient to explain the marked weight loss associated with GLP‐1RA use. Key priorities moving forward are to develop and validate direct measures of food noise, and to conduct mechanistic trials testing whether changes in these pathways actually *drive* reductions in food intake and body weight loss. Longitudinal work is also needed to assess persistence, adaptation, and outcome during dose changes or discontinuation, as well as identification of moderators (e.g., sex, diabetes, eating‐behavior phenotypes, and diet composition) that may explain heterogeneity and inform adjunct strategies.

## Author Contributions

Giorgia Rutigliani, Tzu‐Wen L. Cross, Erica de Jong, Emily S. Glover, Ivan D. Mendoza, Mojisola Adisa, Keigo Tomoo, Xinyi Zhou, and Richard D. Mattes contributed to the conceptualization and writing of the review. All authors contributed to writing, critically reviewed the manuscript for intellectual content, and approved the final version.

## Funding

The authors have nothing to report.

## Conflicts of Interest

The authors declare no conflicts of interest.

## Data Availability

Data sharing not applicable to this article as no datasets were generated or analysed during the current study.
